# Immune thrombocytopenic purpura following mRNA-

**DOI:** 10.15649/cuidarte.3799

**Published:** 2024-07-04

**Authors:** Heiler Lozada-Ramos, Ruth Aralí Martínez-Vega, Liliana Torcoroma-García

**Affiliations:** 1 Universidad de Santander, Facultad de Ciencias Médicas y de la Salud, Instituto de Investigación Masira, Bucaramanga, Colombia. Universidad Santiago de Cali, Escuela de Medicina, Cali, Colombia. E-mail: heiler.lozada00@usc.edu.co Universidad de Santander Universidad de Santander Facultad de Ciencias Médicas y de la Salud Instituto de Investigación Masira Bucaramanga Colombia heiler.lozada00@usc.edu.co; 2 Universidad de Santander, Facultad de Ciencias Médicas y de la Salud, Instituto de Investigación Masira, Bucaramanga, Colombia. E-mail: ruth.martinez@udes.edu.co Universidad de Santander Universidad de Santander Facultad de Ciencias Médicas y de la Salud Instituto de Investigación Masira Bucaramanga Colombia ruth.martinez@udes.edu.co; 3 Universidad de Santander, Facultad de Ciencias Médicas y de la Salud, Instituto de Investigación Masira, Bucaramanga, Colombia. E-mail: l.torcoroma@udes.edu.co Universidad de Santander Universidad de Santander Facultad de Ciencias Médicas y de la Salud Instituto de Investigación Masira Bucaramanga Colombia l.torcoroma@udes.edu.co

**Keywords:** COVID-19, SARS-CoV-2, mRNA Vaccines, Immune Thrombocytopenic Purpura, Platelets, COVID-19, SARS-CoV-2, Vacunas de ARNm, Púrpura Trombocitopénica Autoinmune, Plaquetas, COVID-19, SARS-CoV-2, Vacinas de mRNA, Púrpura Trombocitopênica Autoimune, Plaquetas

## Abstract

**Introduction:**

Herein, it is presented a case report of a Colombian adult male patient, without any previous report of thrombocytopenia or hematological disorders, who developed a mild and chronic paucisymptomatic immune thrombocytopenic purpura, a rare complication following SARS-CoV-2 m-RNA. To the best of our knowledge, this represents the first documented case in Colombia of immune thrombocytopenic purpura associated with mRNA vaccines (BNT162b2 or mRNA-1273), with a comprehensive 2-year clinical follow-up.

**Case Description:**

The patient received the initial and second doses of the mRNA BNT162b2 vaccine in June 2021, the first booster dose in November 2021 (mRNA-1273), and the second booster dose (mRNA BNT162b2) in June 2022. Thrombocytopenia (<100 x109 platelets/L, which is the criterion to define immune thrombocytopenic purpura) was documented after the second vaccination dose and both boosters, and it improved after corticosteroid therapy. However, cycling thrombocytopenia persisted until the clinical follow-up in August 2023, with platelet count ranging from 57 to 191 x109 platelets/L (mean: 103 x109 platelets/L).

**Conclusion:**

Given that secondary immune thrombocytopenic purpura can occur following SARS-CoV-2 mRNA vaccination, systematic research to identify risk factors associated with immune thrombocytopenic purpura due to COVID-19 immunization should be conducted.

## Resumen

**Introducción:**

Este reporte presenta el caso de un paciente colombiano adulto, de sexo masculino, sin antecedentes de trombocitopenia ni trastornos hematológicos, que desarrolló una púrpura trombocitopénica autoinmune paucisintomática leve y crónica, una rara complicación posterior a la vacunación con ARNm contra el virus SARS-CoV-2. Hasta donde se sabe, este es el primer caso documentado en Colombia de púrpura trombocitopénica autoinmune asociada al uso de vacunas de ARNm (BNT162b2 o ARNm-1273), con un seguimiento clínico integral de 2 años.

**Descripción del caso:**

El paciente recibió la primera y segunda dosis de la vacuna de ARNm BNT162b2 en junio de 2021, la primera dosis de refuerzo en noviembre de 2021 (ARNm-1273) y la segunda dosis de refuerzo (ARNm BNT162b2) en junio de 2022. Se documentó trombocitopenia (<100 x109 plaquetas/L, que es el criterio para definir la púrpura trombocitopénica autoinmune) tras la segunda dosis y ambos refuerzos. El paciente mejoró luego del tratamiento con corticosteroides, pero la trombocitopenia cíclica persistió hasta el seguimiento clínico en agosto de 2023, con un recuento de plaquetas que oscilaba entre 57 y 191 x109 plaquetas/L (media: 103x109 plaquetas/L).

**Conclusión:**

Dado que la púrpura trombocitopénica autoinmune puede producirse tras la vacunación con ARNm contra el virus SARS-CoV-2, es necesaria una investigación sistemática para identificar los factores de riesgo asociados a la púrpura trombocitopénica autoinmune debida a la inmunización contra la COVID-19.

## Resumo

**Introdução:**

No presente estudo, é apresentado um relato de caso de um paciente colombiano adulto do sexo masculino, sem qualquer relato anterior de trombocitopenia ou distúrbios hematológicos, que desenvolveu uma púrpura trombocitopênica autoimune leve e crônica paucissintomática, uma complicação rara após a vacinação com mRNA dos vírus SARS-CoV-2. Até onde se sabe, esse é o primeiro caso documentado na Colômbia de púrpura trombocitopênica autoimune associada a vacinas de mRNA (BNT162b2 ou mRNA-1273), com um acompanhamento clínico abrangente de dois anos.

**Descrição do caso:**

O paciente recebeu a dose inicial e a segunda dose da vacina mRNA BNT162b2 em junho de 2021, a primeira dose de reforço em novembro de 2021 (mRNA-1273) e a segunda dose de reforço (mRNA BNT162b2) em junho de 2022. A trombocitopenia (<100 x109 plaquetas/L, que é o critério para definir púrpura trombocitopênica autoimune) foi documentada após a segunda dose de vacinação e ambos os reforços, e melhorou após a terapia com corticosteroides. No entanto, a trombocitopenia cíclica persistiu até o acompanhamento clínico em agosto de 2023, com a contagem de plaquetas variando de 57 a 191 x109 plaquetas/L (média: 103 x109 plaquetas/L).

**Conclusão:**

Dado que a púrpura trombocitopênica autoimune secundária pode ocorrer após a vacinação com mRNA dos vírus SARS-CoV-2, deve ser realizada uma pesquisa sistemática para identificar os fatores de risco associados à púrpura trombocitopênica autoimune devido à imunização contra a COVID-19.

## Introduction

In Colombia, 6,387,837 SARS-CoV-2 infections and 143,113 deaths from COVID-19 have been reported to the National Surveillance System since March 6^th^, 2020, until December 30^th^, 2023 (epidemiological week 52)^1^. In addition, 90.93 million SARS-CoV-2 vaccine doses have been administered from February 17^th^, 2021, to December 31^st^, 2023^2^. In the United States, several cases of autoimmune diseases such as rheumatoid arthritis, bullous pemphigoid, acquired hemophilia A, and immune thrombocytopenia have been registered after vaccination with mRNA vaccines (BNT162b2 or mRNA-1273)^3^^, ^^7^.

Immune thrombocytopenic purpura, also called Immune thrombocytopenia (ITP), is an autoimmune disease caused by antiplatelet autoantibodies. ITP is characterized by low platelet count (<100x109/L) with normal white and red blood cell counts^8^. In these cases, thrombocytopenia can be established as a result of impaired thrombopoiesis, production of platelets with a shortened half-life, or peripheral platelet destruction. The clinical presentation of ITP is widely diverse, ranging from asymptomatic (one-fifth to one-third of the cases are diagnosed by incidental finding of thrombocytopenia) to a severe or lethal condition accompanied by excessive bruising and critical bleeding^9^. This disease is considered a rare disorder that can be primary or secondary to underlying conditions such as systemic pathologies, infections, drugs, or vaccines^10^. Here, a case report of an adult male patient who developed chronic paucisymptomatic or mild ITP secondary to SARS-CoV-2 mRNA vaccines is presented.

## Case Description

A 55-year-old male without any previous reports of thrombocytopenia or other platelet or hematologic disorders is presented. The patient provided written informed consent for the publication of this case report. He lives in Bogotá (Colombia) and works in an office. He has chronic controlled hypothyroidism (diagnosed in 1981) as the only current comorbidity and is under treatment with 50 mcg/day Eutirox®. As for his allergic background, the patient reported having experienced a severe episode of food allergy to shrimp 15 years ago, which has not recurred due to dietary restrictions.

During the COVID-19 emergency, the patient worked from home from March 16th, 2020, to July 12^th^, 2021, and followed the lockdown measures. Before the COVID-19 vaccination, the patient received telemedicine treatment for heartburn, digestive disturbances, and reflux on May 16^th^, 2021. The automated IV-type hemogram showed normal hematological parameters except for a very slight thrombocytopenia [137x109 pj_a_t_e_j_e_t_s_/L. normal range (NR)140-400x109 platelets/L] and an increase in the Medium Platelet Volume (MPV: 12.7 fL; NR: 7.5-11 fL), which were not considered by the medical staff for the installed therapy. The patient was diagnosed with gastroesophageal reflux with esophagitis and dyspepsia (antral gastropathy with erythematous mucosa), which was not related to *Helicobacter pylori* by gastric endoscopy and complementary tests. Antacids and gastroprotective drugs were prescribed before medical discharge. On June 1^st^and June 22^nd^, 2021, the patient received the first and second doses of the anti-SARS-CoV-2 vaccine (mRNA BNT162b2, Pfizer-BioNTech) without any immediately apparent significant adverse effects, such as fever, arthralgia, myalgias, or other symptoms reported by the patient. However, a platelet count of 110x109 platelets/L was documented on June 22^nd^, 2021.

On June 28^th^, 2021, the patient tested positive for SARS-CoV-2 infection by RT-qPCR test, remaining asymptomatic until two weeks after the onset of infection when he sought medical attention due to paroxysmal night-time hyperthermia sensation (afebrile) and diaphoresis. No manifest alterations were recorded in general appearance, vital signs (normal temperature, oxygen saturation, and arterial blood pressure), and physical examination (Body Mass Index: 25.7). The hemogram test showed moderate thrombocytopenia (July 15^th^, 2021: 83x109 platelets/L) without any significant difference between citrate and Ethylenediaminetetraacetic acid (EDTA) counts by manual or automated methods. Abundant macro-platelets (>50%, NR: 0.5-5.0%) were observed in the peripheral blood smear (PBS), elevated MPV (12.7 fL), neutropenia (1.5x109 neutrophils/L), leukopenia (4.0x103 leukocytes/pL), and a decrease in the Neutrophil/Lymphocyte Index (NLI: 0.7; NR: 0.88-4.0). Mild diffuse hepatic steatosis was diagnosed by hepatobiliary ultrasound. Kidney and thyroid function parameters were found to be within the normal range. Oral prednisolone (35 mg/day) was administered for seven continuous days, after which the platelet counts recovered to pre-vaccination levels (139x109 platelets/L).

On November 5^th^, 2021, the patient underwent complementary blood tests which showed negative results for human immunodeficiency virus, hepatitis B virus, hepatitis C virus, and syphilis infections as well as for autoantibodies (anti-Sjogren’s syndrome-related antigen A, anti-small nuclear riboproteins, anti-neutrophil cytoplasm, anti-phospholipids, thyroid peroxidase antibodies, and rheumatoid factor). Normal profiles were observed in vitamins (B12, B9, and D vitamins), coagulation, and platelet aggregation parameters. Only antinuclear antibodies (ANAs) (1:160 with fine speckled pattern AC-4) and Coombs tests were outside the normal parameters. A myelogram demonstrated medullar hypoplasia with megaloblastic erythroid changes, bicytopenia (thrombocytopenia and grade I neutropenia), without dysplasia and neoplastic infiltrate. As therapy, oral doses of Azatriopine (150 mg/day for 60 days) were administered. This therapy was suspended after a week due to significant exacerbation of leukopenia (3.0x109 leukocytes/L), neutropenia (1.0x109 neutrophils/L), and thrombocytopenia (November 24^th^, 2021: 52x109 platelets/L). Spontaneous normalization of the hematological parameters was observed within a week, except for thrombocytopenia (116x10^9^ platelets/L).

On November 27^th^, 2021, the patient received the first booster dose (mRNA-1273, Moderna). Six days after vaccination, the platelet count newly declined (December 2^nd^, 2021: 87x109 platelets/L), as well as the NLI (0.82). On February 7^th^, 2022, the patient had SARS-CoV-2 infection for the second time. Unlike the first asymptomatic infection, the patient reported abdominal pain, diarrhea, fever, cough, rhinorrhea, headache, asthenia, and adynamia, and the platelet count slightly decreased (February 25^th^, 2022: 141x109 platelets/L).

On June 5^th^, 2022, the patient received the second booster dose (mRNA BNT162b2, Pfizer-BioNTech). After two weeks, the patient consulted by acute symptoms of cough, rhinorrhea, headache, and asthenia (negative RT-qPCR for SARS-CoV-2), and the platelet count was 93x109 platelets/L on June 25^th^, 2022. Likewise, since June 2021, the patient reported a persistent facial sensation of paroxysmal hyperthermia (afebrile) and diaphoresis at night. Additionally, the esophagitis symptoms were exacerbated, for which endoscopy and biopsy analysis were performed, revealing eosinophilic esophagitis. These results were accompanied by an elevation of total immunoglobulin E levels (321 UI/mL, NR <100 UI/mL), mild thrombocytopenia (105x109 platelets/L), and macro-platelets (MPV: 12.2 fL). D-dimer (700 ng/mL), ferritin (379 ng/dL), and fibrinogen (455 mg/dL) increased. Dexamethasone (16 mg/day) was prescribed for three continuous days. After medication and during the next three months, normalization of all hemogram parameters was observed, including platelets (191x109 platelets/L). Likewise, d-dimer, ferritin, and fibrinogen were restored to normal ranges.

From September 2022 to August 2023, the patient presented platelet count fluctuations (mean: 103x109 platelets/L; range: 57-191x109 platelets/L). Normal or almost normal platelets count (range: 139-193x109 platelets/L) were observed only post-corticoid therapy and remained stable for two or three months after this treatment.

**Figure 1:**
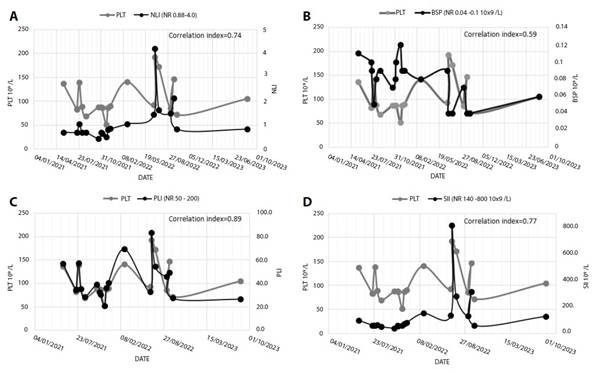
Correlation between thrombocytopenia and hematological inflammatory parameters.

In the PBS, a high percentage of macro-platelets (>50%) was always present, which was accompanied by elevated MPV (mean: 12.5 fL; range: 11-13.4 fL). The thrombocytopenia episodes presented a significant correlation with lower values of NLI (mean: 1.11; range: 0.45-4.2; correlation index: 0.74; Figure 1A), as well as with lower Platelet/Lymphocyte index (PLI) (mean: 42; range: 21-83; NR: 50 200; correlation index: 0.89; Figure 1C). These indexes presented an inversed behavior with general inflammatory biomarkers (reactive-C-protein, d-dimer, fibrinogen, and ferritin), which evidenced an increase during the thrombocytopenia crisis. No significant correlation was observed with the absolute basophil counts (correlation index: 0.59; Figure 1B). Interestingly, a directly proportional relationship was observed between platelet count and the Systemic Immune-Inflammation index (SII) (thrombocytopenia were accompanied by low SII; correlation index: 0.77; Figure 1D).

*A) Platelets count (PLT) and Neutrophil/Lymphocyte Index (NLI); B) Platelets count (PLT) and basophilic (BSP) count correlation; C) Platelets count (PLT) and Platelet/Lymphocyte Index (PLI); and D) Platelets count (PLT) and Systemic Immune-inflammation index (SII). NR: Normal range*

From June 2021 to August 2023, the total serum immunoglobulin E levels remained elevated (>300 UI/L). Coherently, during this time, the patient presented exacerbation of allergic manifestations such as persistent rhinitis and flu-like symptoms. Taking into consideration the clinical backgrounds and clinical findings observed after COVID-19 vaccination, the patient was diagnosed with ITP secondary to anti-SARS-CoV-2 immunization without hemorrhagic or thrombotic phenotype. Currently, the patient is under observation every three months by a specialized hematological consultant and is receiving symptomatic treatment for persistent esophagitis and rhinitis. The dataset is available in the public database manager Mendeley Data^11^.

## Discussion

Today, the crucial role that collective mass vaccination efforts played in safeguarding human lives and averting further social and economic global devastation caused by COVID-19 is indisputable^12^. In this scenario, new platforms of third-generation mRNA vaccines were developed, studied, and simultaneously administered under emergency conditions without enough data provided by previous clinical trials. Over traditional vaccines, these technological approaches have demonstrated advantages in design and production^13^. However, there are many safety challenges and unexpected adverse effects to SARS-CoV-2 mRNA vaccines that constitute a major concern in public health and require comprehensive monitoring and adequate study^14^.

This case report aims to contribute to COVID-19 vaccine surveillance. It presents a newly diagnosed case of ITP, classified according to the disease phase classification^10^, where thrombocytopenia was observed within the first three weeks after receiving the first dose of the mRNA BNT162b2 vaccine. In this regard, although the patient had slight thrombocytopenia before the COVID-19 vaccination, platelet counts were higher than 100x109 platelets/L, which is the threshold level to consider ITP^10^. In general, ITP is a rare condition with an annual incidence of fewer than 5 cases per 100,000 inhabitants^15^. However, its incidence has been diagnosed more frequently since the implementation of massive vaccination programs against SARS-CoV-2^16^. In this regard, autoimmune and immune cross-reactions such as ITP induced by vaccination have been reported, especially associated with mRNA-COVID-19 vaccines14,17,18.

In the presented case, post-vaccinal ITP was associated with both vaccine brands, BNT162b2 mRNA (first and second doses and second boosted doses) and mRNA-1273 (first boosted doses). This combined scheme was commonly applied in Colombia for young adult populations. Both vaccines have demonstrated very potent efficacy in generating neutralizing antibodies against SARS-CoV-2. Nevertheless, this same immune-responsive capability may also be correlated with a cross-reactive response against thrombocytes^19^. The etiology of vaccine-associated ITP has been hypothesized to be related to antiplatelet autoantibodies^19^. However, this kind of antibody was not detected in our patient. Only a slight positive result was observed for ANAs and Coombs tests. These results are coherent with previous studies that reported a low percentage of patients diagnosed with COVID-19 vaccine-associated ITP were positive for antiplatelet autoantibodies^17^.

In this case, the persistence of circulant macro-platelets in elevated percentages (>50%) was reported after COVID-19 vaccination and until the final observation period (August 2023). This finding can be associated with high peripheral platelet destruction by an immune-mediated process and the concomitant increase of thrombopoiesis as a compensatory mechanism. In this regard, macro platelets are young thrombocytes characterized to be highly granulated and more reactive^20^.

It is important to mention that our patient reported a familial background of several autoimmune diseases, such as Hashimoto’s thyroiditis, pernicious anemia, psoriasis, asthma, rhinitis, and diverse cutaneous allergies. As a personal background, the patient reported chronic hypothyroidism (since adolescence) and gastric problems. The latter was exacerbated after 15 days of COVID-19 immunization, and further clinical analysis revealed a diagnosis of eosinophilic esophagitis, which is an immunogenic process triggered by an allergic response to several antigens and leads to inflammation of the esophageal mucosa. Regarding that, repeated antigenic exposure can lead to the recruitment of eosinophils in the esophagus, addressing the remodeling and fibrosis of the inflamed tissue^21^. This disease was associated with concomitant immunoglobulin E sensitization (elevated IgE levels) to an unknown allergen.

The COVID-19 vaccine-associated ITP case reported herein evidenced benign clinical presentation (platelet count near 100x109/L in convalescence), and it was treatment responsive with high-dose corticosteroids since normal or almost normal platelets count (140-190x109 platelets/L) were observed temporarily (during two or three months) only post-therapy. This clinical behavior is compatible with previous observations obtained from a systematic analysis of COVID-19 vaccine-associated ITP cases, in which the disease had a mild or asymptomatic presentation accompanied by a generally good response to conventional ITP therapy^17^. In our patient, thrombocytopenia cycles were always correlated with lowered NLI, PLI, and SII. These indices have been used as indicators in the prognosis and progression of autoimmune diseases such as ITP. In agreement with the observed behavior of PLI reported herein, this index was significantly lower in relapsed ITP, with a significant correlation between PLI and platelet count and a negative correlation between PLI and lymphocyte count^22^. Likewise, an improvement in the PLI value indicated a positive response to ITP therapy, and declined PLI was a predictor of relapse and glucocorticoid resistance^23^. In addition, PLI was an independent variable for the direct risk of chronic and recurrent ITP^24^.

## Conclusion

In the reported case, the thrombocytopenia persisted and progressed to a chronic non-bleeding condition two years after the first vaccine dose administration. During this period, a few episodes of temporary recovery of platelet counts (within the reference range) were observed. According to the available 2-year follow-up data, the patient also reported persistence of paroxysmal at night face hyperthermia sensation (afebrile) and diaphoresis. Considering that secondary ITP and other hematological disorder incidence have significantly increased due to COVID-19 vaccination and mRNA vaccines, a post-vaccination hemogram check could be a useful and inexpensive follow-up measure before the administration of second or booster doses. Likewise, it is important to carry out systematic research to identify individual or vaccine risk factors associated with ITP presentation as a consequence of COVID-19 immunization.
